# Knowledge of Glasgow Coma Scale among Nurses in a Tertiary Care Centre: A Descriptive Cross-sectional Study

**DOI:** 10.31729/jnma.7673

**Published:** 2022-08-31

**Authors:** Bidur KC, Mohamed Zaidan Adil

**Affiliations:** 1 Department of Neurosurgery, Kathmandu Medical College and Teaching Hospital, Sinamangal, Kathmandu, Nepal

**Keywords:** *glasgow coma scale*, *knowledge*, *nurses*, *tertiary care centre*

## Abstract

**Introduction:**

Glasgow Coma Scale is a dependable and unprejudiced neurological evaluation kit applied for evaluating and recording the level of consciousness of a person. Evaluation of consciousness level using Glasgow Coma Scale is a tool necessitating knowledge which is vital in identifying immediate worsening of level of consciousness. Critical thinking used with skill and knowledge in evaluating Glasgow Coma Scale is the groundwork of nursing practice to avoid delay in clinical worsening and treatment. The aim of this study was to find out the prevalence of inadequate knowledge of Glasgow Coma Scale among nurses working in a tertiary care centre.

**Methods:**

A descriptive cross-sectional study was performed among registered nurses working in different wards and Intensive Care Unit at tertiary care centre between 1 June 2022 and 30 June 2022 after receiving ethical approval from the Institutional Review Committee (Reference number: 2905202211). Convenience sampling was done. Self-administered structured questionnaires were used to collect data to assess the knowledge of Glasgow Coma Scale. Point estimate and 95% Confidence Interval were calculated.

**Results:**

Among 91 nurses, inadequate knowledge of the Glasgow Coma Scale was found in 48 nurses (52.70%) (42.30-63.10, 95 % Confidence Interval).

**Conclusions:**

The prevalence of inadequate knowledge of the Glasgow Coma Scale among nurses was found to be similar when compared to other studies done in similar settings.

## INTRODUCTION

In 1974, Teasdale and Jennett, innovated Glasgow Coma Scale (GCS) to objectively quantify coma severity in acute medical and trauma patients.^1^ Beside trauma, GCS is utilised to assess clinical care of literally any disease, be it infective, degenerative, metabolic or neoplastic.^2^ GCS continues to be the benchmark for evaluation, continuous surveillance, prognosis and clinical decision about consciousness in such patients.^3,4^

Versatile use of GCS across different clinical scenario mandates all nurses to be knowledgeable about it, as they may require to conduct GCS at any point of time.^1^ Swift and accurate evaluation will reduce neurological complications, inaccurate diagnostic procedures, morbidity and mortality.^5^ Moreover, evaluation errors are hazardous and have potentially irreversible negative impact paired with worse outcomes because of the inability to recognize or manage complications in a prompt and fruitful manner.^6^

This study aims to find out the prevalence of inadequate knowledge of GCS among nurses in a tertiary care centre.

## METHODS

A descriptive cross-sectional study was performed at Kathmandu Medical College Teaching Hospital (KMCTH), Sinamangal, Kathmandu between 1 June 2022 and 30 June 2022. This study was approved by the Institutional Review Committee (IRC) of KMCTH (Reference number: 2905202211). Informed consent was taken from the registered nurse of wards and the intensive care unit (ICU) to enrol in the study. Registered nurses who were working in the medical, and surgical wards and different intensive care units of KMCTH were enrolled into the study. Those who were on leave during the data collection period were excluded. Convenience sampling was done, and the sample size was calculated using the formula:


$$\begin{array}{ll} \text{n} & ={\text{Z}}^{2}\times \frac{\text{p}\times \text{q}}{{\text{e}}^{\text{2}}}\\ & =1.96^{2}\times \frac{0.65\times 0.35}{0.10^{2}}\\ & =88 \end{array}$$

Where,

n= required sample size
Z= 1.96 at 95% Confidence Interval (CI)
p= prevalence of inadequate knowledge about GCS assessment, 65.70%^7^
q= 1-p
e= margin of error, 10%

However, 91 samples were taken in this study. Questionnaires were utilised to collect data regarding knowledge of GCS among nurses. Questionnaires were adapted from a previous study by Shoqirat and Waterhouse in a multiple-choice question (MCQ) format.^8,9^ They were asked to complete it independently within 15 to 20 minutes under the surveillance of the researcher. Questionnaires consisted of two parts. The first part consists of sociodemographic data in terms of age, gender, level of education, current wards or ICU and years of experience. The second part includes 15 MCQs to assess the knowledge of GCS among nurses whereby only one answer is correct in each MCQ. Each question was scored as 1 mark if the answer was correct and as 0 if the answer was incorrect. Knowledge of GCS comprises a total score of 15. The level of knowledge was further categorised into good knowledge when the score was between 12 to 15, average knowledge when the score was between 9 to 11 and poor knowledge when the score was 0 to 8. The collected data were entered and analysed in IBM SPSS Statistics 25.0. Point estimate and 95% CI were calculated.

## RESULTS

Among a total of 91 nurses, inadequate knowledge of the Glasgow Coma Scale was found in 48 nurses (52.70%) (42.30-63.10, 95% CI). The age of the nurses ranged from 19 to 35 years with a mean age of 24.48±3.33 years. The maximum number of age groups was between 19 to 25 years. Years of experience of the nurses ranged from 1 month to 14 years with a mean of 3.21±2.96 years (Table 1).

**Table 1:** Socio-demographic characteristics of nurses with a poor level of knowledge of GCS (n= 48).

Variables	n (%)
**Age in years**	
19-25	35 (72.92)
26-30	11 (22.91)
31-35	2 (4.17)
**Academic qualification**	
Proficiency Certificate level nursing	16 (33.33)
Bachelor of nursing	32 (66.67)
**Current department**	
Medicine ward	20 (41.67)
Surgery ward	12 (25.00)
Surgery ICU	10 (20.83)
Neurosurgery ICU	6 (12.50)
**Year of experience**	
≤1	15 (31.25)
1.1-3	12 (25.00)
3.1-5	14 (29.17)
>5	7 (14.58)
Formal training on GCS	-

## DISCUSSION

The Usage of GCS became further popular in the 1980s when it was advocated by the first edition of the Advanced Trauma and Life Support for utilisation in every trauma patient, and the World Federation of Neurosurgical Societies also used this tool for grading subarachnoid haemorrhage.^1^ The GCS is commonly utilised for field triage verdicts, including emergency treatment and finding out appropriate destinations for shifting of patients. Serial GCS evaluation also has a role in the clinical course of a patient.^1^ Afterall GCS is a priceless tool to obviate delay in recognizing clinical worsening of patients; therefore poor level of knowledge among nurses could negatively impact on safety and prognosis of the patients.

This study demonstrated poor level of knowledge in 52.70%. This means the majority of nurses showed an inadequate level of knowledge. This is comparable to the findings done in Malaysia found that 55.56% had poor knowledge on GCS.^10^ Similarly, Study done in Ghana found that a little more than half of the participants (50.40%) had poor knowledge of the GCS as a whole.^11^ Likewise, Study performed in Lahore concluded that the majority of nurses (65.70%) have inadequate knowledge and weak evaluation regarding GCS.^7^ In addition, research done in Vietnamese nurses revealed that 52.10% of the nurses were shortage of essential knowledge about GCS mostly when it comes to the clinical background, only very few nurses (13.30%) pointed that their existing knowledge on the implementation of GCS is adequate.^12^ Furthermore, study performed in Baghdad found that the nurses have insufficient knowledge regarding all items related to GCS.^13^

Mean age of the nurses with poor level of knowledge were 24.48±3.33 years in the present study. Identical to this study, a study conducted in Jordan and Nepal observed mean age of 26.30±80 years and 23.24±2.66 years respectively.^5,14^ All the participants with poor level of knowledge in the current study were female. Female preponderance with 90.40% each was also seen in both the studies performed in Singapore.^15,16^ In this study 66.67% of nurses with poor level of knowledge had completed their bachelor in nursing. Identical to this study, the majority of nurses (89.80%) have done post graduation degree in a study done in Brazil.^17^ Mean duration of year of experience was 3.21±2.96 years in present study. Previous study from Nepal also demonstrated 89.60% of nurses had less than or equal to 3 years of experience.^14^ None of the nurses with poor level of knowledge took formal training on GCS in current study. In concordance with this study, study in Nigeria observed none of respondents received further training on GCS.^18^

Nurses in ICU are needed to execute monitoring and evaluation of GCS on a more repeated manner as opposed to nurses in general wards. With the repeated use of the tool in day to day manner, they acquire an intuition on its implementation in patients with various types of diseases involving central nervous system, that may not be accomplished by nurses who are working in general wards.^19,20^ In this study also nurses of neurosurgery ICU demonstrated lowest poor level of knowledge whereas nurses from medical ward showed highest poor level of knowledge. In concordance with this study, study done in Vietnam reported neurosurgery ICU had a higher mean score on knowledge as compared to the other units.^12^ Previous studies done in Singapore showed that healthcare workers working in the ICUs have the highest knowledge of GCS.^15^ Moreover, this is also in harmony with the study done in Edinburgh, demonstrated that those student nurses who are working in neuroscience wards had a better understanding of the GCS in comparison with their peers who did not undertake such attachment.^8^

Mean score of the whole sample out of 15 questionnaires for assessment of level of knowledge was 8.16±1.93 in this study. Study conducted in Jordan also reported a low level of knowledge with a mean score of 7.38±1.96 using the same questionnaire.^5^ Limitation of present study is that it was conducted in a single centre with small sample size.

## CONCLUSIONS

In this study, the prevalence of inadequate knowledge was found to be similar to other studies done in similar settings. Nurses from Neurosurgery ICU demonstrated the lowest whereas the medical ward showed the highest poor level of knowledge. There should be a structured education and frequent training programme along with a demonstration on the use of GCS so that nurses can accurately monitor the level of consciousness, thereby ensuring the patient's safety.
